# Supplementary material for: Aerobic exercise and brain structure among military service members and Veterans with varying histories of mild traumatic brain injury: A LIMBIC-CENC exploratory investigation
Source: PLoS One. 2025 Mar 31;20(3):e0320004. doi: 10.1371/journal.pone.0320004 (PMC11957293; doi:10.1371/journal.pone.0320004)
Supplement: S1 Supplemental Materials — Also contains “Supplemental Table 1. Gray matter variables from FreeSurfer v7.1.1 processing” detailing all individual region of interest labels and formulas for aggregate regionns of interest based on those labels. (DOCX) [file pone.0320004.s001.docx]

**S1. Supplemental Materials**

**Supplemental Methods.** Comparison of demographic, MVAE, and mTBI characteristics between those with MRI data available for this study (*n* = 1,349) vs. those without (*n* = 902).

· **Age**

o Mean age (standard deviation)

§ With MRI data = 41.4 (10.3) years; without MRI data = 42.4 (10.2) years

· Independent samples *t*-test results: *t* = 2.344; *p* = 0.02; Cohen’s *d* (95% CI) = 0.10 (.02 - .18).

§ Comment

· Statistically significant, but not clinically meaningful difference based on the mean age difference between groups (1 year). Results were nearly identical based on equal variances assumed or not assumed (tested via Levene’s test).

· **Sex**

o Proportion of females and males, *n* (%)

§ With MRI data = females 174 (12.9%), males 1175 (87.1%); without MRI data = females 109 (11.9%), males 804 (88.0%)

· Chi-squared proportional analysis: *X*^2^(df=1) = 0.458; *p* = 0.50; Cramer’s *V* = 0.014.

· **mTBI Group (*0 mTBI; 1-2 mTBI; 3+ mTBI*)**

o Proportion of those in each mTBI group with and without MRI data, *n* (%)

§ With MRI data = 0 mTBI 260 (19.3%), 1-2 mTBI 636 (47.1%), 3+ mTBI 453 (33.6%); without MRI data = 0 mTBI 148 (16.2%), 1-2 mTBI 441 (48.2%), 3+ mTBI 325 (35.6%)

· Chi-squared proportional analysis: *X*^2^(df=2) = 3.628; *p* = 0.16; Cramer’s *V* = 0.04.

· **MVAE participation group (*Inactive; Insufficiently Active; Active; Highly Active*)**

o Proportion of those in each MVAE group with and without MRI data, *n* (%)

§ With MRI data = Inactive 457 (33.9%), Insufficiently Active 429 (31.8%), Active 238 (17.6%), Highly Active 225 (16.7%); without MRI data = Inactive 308 (33.7%), Insufficiently Active 283 (31.0%), Active 152 (16.6%), Highly Active 159 (17.4%).

· Chi-squared proportional analysis: *X*^2^(df=3) = 0.523; *p* = 0.91; Cramer’s *V* = 0.02.

· **Education (*high school or less*; *some college or technical school*; *college degree or higher*)**

o Proportion of those in each education attainment group with and without MRI data, *n* (%)

§ With MRI data = high school 169 (12.5%), some college 539 (40.0%), college degree 641 (47.5%); without MRI data = high school 116 (12.7%), some college 364 (39.8%), college degree 433 (47.4%).

· Chi-squared proportional analysis: *X*^2^(df=2) = 0.0.16; *p* = 0.99; Cramer’s *V* = 0.003.

· **Current vs. former military status**

o Proportion of those in each duty status group (*active*; *Veteran*) with and without MRI data, *n* (%)

§ With MRI data = active 238 (17.6%), Veteran 1.111 (82.4%); without MRI data = active 183 (20.0%), Veteran 725 (79.3%).

· Chi-squared proportional analysis: *X*^2^(df=1) = 2.256; *p* = 0.13; Cramer’s *V* = 0.03.

**Among those with TBI history**

· **Interaction with blast TBI (any vs. none)**

o Proportion of those with and without MRI data, *n* (%)

§ With MRI data = one or more blast-related mTBI 476 (43.7%), no blast-related mTBI 613 (56.3%); without MRI data = one or more blast-related mTBI 372 (48.6%), no blast-related mTBI 393 (51.3%).

· Chi-squared proportional analysis: *X*^2^(df=1) = 4.378; *p* = 0.04; Cramer’s *V* = .05.

§ Comment

· 5% difference between MRI groups for those with/without exposure to blast-related mTBI; effect size (*V*) was negligible.

· **Time since most recent TBI**

o Mean number of years (standard deviation)

§ With MRI data = 11.5 (9.2) years; without MRI data = 12.3 (10.3) years

· Independent samples *t*-test results: *t* = 1.795; *p* = 0.07; Cohen’s *d* (95% CI) = 0.09 (.01 - .18).

**Supplemental Table 1. Gray matter variables from FreeSurfer v7.1.1 processing.** Individual region of interest labels and formulas for aggregate regions of interest (highlighted in gold) based on those labels.

| **Variable description** | **Formula** |
| --- | --- |
| Subject identification number |  |
| Estimated total intracranial volume |  |
| Total brain segmentation (not ventricle) volume |  |
| Total ventricular volume | = [scv_l_lv + scv_l_ilv + scv_3v + scv_4v + scv_l_cp + scv_r_lv + scv_r_ilv + scv_r_cp + scv_5v] |
| Ventricle to brain ratio | = [(tbv / tvv) * 100] |
| Composite volume of the left frontal lobe | = [gmv_l_cmf + gmv_l_lof + gmv_l_mof + gmv_l_ifop + gmv_l_iforb + gmv_l_iftri + gmv_l_prec + gmv_l_rmf + gmv_l_sf + gmv_l_fpole] |
| Volume of the left caudal middle frontal gyrus |  |
| Volume of the left lateral orbitofrontal gyrus |  |
| Volume of the left medial orbitofrontal gyrus |  |
| Volume of the left pars opercularis |  |
| Volume of the left pars orbitalis |  |
| Volume of the left pars triangularis |  |
| Volume of the left precentral gyrus |  |
| Volume of the left rostral middle frontal gyrus |  |
| Volume of the left superior frontal gyrus |  |
| Volume of the left frontal pole |  |
| Composite volume of the left temporal lobe | = [gmv_l_bsts + gmv_l_ent + gmv_l_fus + gmv_l_it + gmv_l_mt + gmv_l_phc + gmv_l_st + gmv_l_tpole + gmv_l_tt] |
| Volume of the left bank of the superior temporal sulcus |  |
| Volume of the left entorhinal cortex |  |
| Volume of the left fusiform gyrus |  |
| Volume of the left inferior temporal gyrus |  |
| Volume of the left middle temporal gyrus |  |
| Volume of the left parahippocampal gyrus |  |
| Volume of the left superior temporal gyrus |  |
| Volume of the left temporal pole |  |
| Volume of the left transverse temporal gyrus |  |
| Volume of the left insular cortex |  |
| Composite volume of the left cingulate cortex | = [gmv_l_cacc + gmv_l_icc + gmv_l_pcc + gmv_l_racc] |
| Volume of the left caudal anterior cingulate cortex |  |
| Volume of the left isthmus of the cingulate cortex |  |
| Volume of the left posterior cingulate cortex |  |
| Volume of the left rostral anterior cingulate cortex |  |
| Composite volume of the left parietal lobe | = [gmv_l_ip + gmv_l_parac + gmv_l_postc + gmv_l_precun + gmv_l_sp + gmv_l_sm] |
| Volume of the left inferior parietal gyrus |  |
| Volume of the left paracentral gyrus |  |
| Volume of the left postcentral gyrus |  |
| Volume of the left precuneus |  |
| Volume of the left superior parietal gyrus |  |
| Volume of the left supramarginal gyrus |  |
| Composite volume of the left occipital lobe | = [gmv_l_cun + gmv_l_lo + gmv_l_ling + gmv_l_pericalc] |
| Volume of the left cuneus |  |
| Volume of the left lateral occipital gyrus |  |
| Volume of the left lingual gyrus |  |
| Volume of the left pericalcarine gyrus |  |
| Composite volume of the right frontal lobe | = [gmv_r_cmf + gmv_r_lof + gmv_r_mof + gmv_r_ifop + gmv_r_iforb + gmv_r_iftri + gmv_r_prec + gmv_r_rmf + gmv_r_sf + gmv_r_fpole] |
| Volume of the right caudal middle frontal gyrus |  |
| Volume of the right lateral orbitofrontal gyrus |  |
| Volume of the right medial orbitofrontal gyrus |  |
| Volume of the right pars opercularis |  |
| Volume of the right pars orbitalis |  |
| Volume of the right pars triangularis |  |
| Volume of the right precentral gyrus |  |
| Volume of the right rostral middle frontal gyrus |  |
| Volume of the right superior frontal gyrus |  |
| Volume of the right frontal pole |  |
| Composite volume of the right temporal lobe | = [gmv_r_bsts + gmv_r_ent + gmv_r_fus + gmv_r_it + gmv_r_mt + gmv_r_phc + gmv_r_st + gmv_r_tpole + gmv_r_tt] |
| Volume of the right bank of the superior temporal sulcus |  |
| Volume of the right entorhinal cortex |  |
| Volume of the right fusiform gyrus |  |
| Volume of the right inferior temporal gyrus |  |
| Volume of the right middle temporal gyrus |  |
| Volume of the right parahippocampal gyrus |  |
| Volume of the right superior temporal gyrus |  |
| Volume of the right temporal pole |  |
| Volume of the right transverse temporal gyrus |  |
| Volume of the right insular cortex |  |
| Composite volume of the right cingulate cortex | = [gmv_r_cacc + gmv_r_icc + gmv_r_pcc + gmv_r_racc] |
| Volume of the right caudal anterior cingulate cortex |  |
| Volume of the right isthmus of the cingulate cortex |  |
| Volume of the right posterior cingulate cortex |  |
| Volume of the right rostral anterior cingulate cortex |  |
| Composite volume of the right parietal lobe | = [gmv_r_ip + gmv_r_parac + gmv_r_postc + gmv_r_precun + gmv_r_sp + gmv_r_sm] |
| Volume of the right inferior parietal gyrus |  |
| Volume of the right paracentral gyrus |  |
| Volume of the right postcentral gyrus |  |
| Volume of the right precuneus |  |
| Volume of the right superior temporal gyrus |  |
| Volume of the right supramarginal gyrus |  |
| Composite volume of the right occipital lobe | = [gmv_r_cun + gmv_r_lo + gmv_r_ling + gmv_r_pericalc] |
| Volume of the right cuneus |  |
| Volume of the right lateral occipital gyrus |  |
| Volume of the right lingual gyrus |  |
| Volume of the right pericalcarine gyrus |  |
| Subcortical volume of the left lateral ventricle |  |
| Subcortical volume of the left inferior lateral ventricle |  |
| Subcortical volume of the left cerebellum white matter |  |
| Subcortical volume of the left cerebellum cortex |  |
| Subcortical volume of the left caudate |  |
| Subcortical volume of the left putamen |  |
| Subcortical volume of the left pallidum |  |
| Subcortical volume of the third ventricle |  |
| Subcortical volume of the fourth ventricle |  |
| Subcortical volume of the CSF |  |
| Subcortical volume of the left nucleus accumbens area |  |
| Subcortical volume of the left ventral diencephalon |  |
| Subcortical volume of the left vessel |  |
| Subcortical volume of the left choroid plexus |  |
| Subcortical volume of the right lateral ventricle |  |
| Subcortical volume of the right inferior lateral ventricle |  |
| Subcortical volume of the right cerebellum white matter |  |
| Subcortical volume of the right cerebellum cortex |  |
| Subcortical volume of the right caudate |  |
| Subcortical volume of the right putamen |  |
| Subcortical volume of the right pallidum |  |
| Subcortical volume of the right nucleus accumbens area |  |
| Subcortical volume of the right ventral diencephalon |  |
| Subcortical volume of the right vessel |  |
| Subcortical volume of the right choroid plexus |  |
| Subcortical volume of the fifth ventricle |  |
| Subcortical volume of the optic chiasm |  |
| Subcortical volume of the total corpus callosum | = [scv_scc + scv_pmbcc + scv_mbcc + scv_ambcc + scv_gcc] |
| Subcortical volume of the splenium of the corpus callosum |  |
| Subcortical volume of the posterior midbody of the corpus callosum |  |
| Subcortical volume of the midbody of the corpus callosum |  |
| Subcortical volume of the anterior midbody of the corpus callosum |  |
| Subcortical volume of the genu of the corpus callosum |  |
| Subcortical volume of the total brain segmentation |  |
| Subcortical volume of the total brain segmentation (not ventricles) |  |
| Subcortical volume of the left hemisphere cortex |  |
| Subcortical volume of the right hemisphere cortex |  |
| Subcortical volume of the total cortex |  |
| Subcortical volume of the left cerebral white matter |  |
| Subcortical volume of the right cerebral white matter |  |
| Subcortical volume of the total cerebral white matter |  |
| Subcortical volume of the total subcortical gray matter |  |
| Subcortical volume of the total gray matter |  |
| Subcortical volume of the total supratentorial region |  |
| Subcortical volume of the total voxels of the supratentorial region |  |
| Subcortical volume of the total brain mask |  |
| Subcortical volume of the total brain segmentation-to-eTIV ratio |  |
| Subcortical volume of the total brain mask-to-eTIV ratio |  |
| Subcortical volume of the left surface holes |  |
| Subcortical volume of the right surface holes |  |
| Subcortical volume of all white matter hypointensities |  |
| Structural volumes used for segmentation [0 = T1 only \| 1 = T1+T2] |  |
| Hippocampal subfield volume of the left hippocampal tail |  |
| Hippocampal subfield volume of the left subiculum body |  |
| Hippocampal subfield volume of the left CA1 body |  |
| Hippocampal subfield volume of the left subiculum head |  |
| Hippocampal subfield volume of the left hippocampal fissure |  |
| Hippocampal subfield volume of the left presubiculum head |  |
| Hippocampal subfield volume of the left CA1 head |  |
| Hippocampal subfield volume of the left presubiculum body |  |
| Hippocampal subfield volume of the left parasubiculum |  |
| Hippocampal subfield volume of the left molecular layer HP head |  |
| Hippocampal subfield volume of the left molecular layer HP body |  |
| Hippocampal subfield volume of the left GC-ML-DG body |  |
| Hippocampal subfield volume of the left CA3 body |  |
| Hippocampal subfield volume of the left GC-ML-DG body |  |
| Hippocampal subfield volume of the left CA4 head |  |
| Hippocampal subfield volume of the left CA4 body |  |
| Hippocampal subfield volume of the left fimbria |  |
| Hippocampal subfield volume of the left CA3 head |  |
| Hippocampal subfield volume of the left HATA |  |
| Hippocampal subfield volume of the left whole hippocampal body |  |
| Hippocampal subfield volume of the left whole hippocampal head |  |
| Hippocampal subfield volume of the left whole hippocampus hc |  |
| Hippocampal subfield volume of the right hippocampal tail |  |
| Hippocampal subfield volume of the right subiculum body |  |
| Hippocampal subfield volume of the right CA1 body |  |
| Hippocampal subfield volume of the right subiculum head |  |
| Hippocampal subfield volume of the right hippocampal fissure |  |
| Hippocampal subfield volume of the right presubiculum head |  |
| Hippocampal subfield volume of the right CA1 head |  |
| Hippocampal subfield volume of the right presubiculum body |  |
| Hippocampal subfield volume of the right parasubiculum |  |
| Hippocampal subfield volume of the right molecular layer HP head |  |
| Hippocampal subfield volume of the right molecular layer HP body |  |
| Hippocampal subfield volume of the right GC-ML-DG body |  |
| Hippocampal subfield volume of the right CA3 body |  |
| Hippocampal subfield volume of the right GC-ML-DG body |  |
| Hippocampal subfield volume of the right CA4 head |  |
| Hippocampal subfield volume of the right CA4 body |  |
| Hippocampal subfield volume of the right fimbria |  |
| Hippocampal subfield volume of the right CA3 head |  |
| Hippocampal subfield volume of the right HATA |  |
| Hippocampal subfield volume of the right whole hippocampal body |  |
| Hippocampal subfield volume of the right whole hippocampal head |  |
| Hippocampal subfield volume of the right whole hippocampus hc |  |
| Structural volumes used for segmentation [0 = T1 only \| 1 = T1+T2] |  |
| Amygdalar nuclei volume of the left lateral nucleus |  |
| Amygdalar nuclei volume of the left basal nucleus |  |
| Amygdalar nuclei volume of the left accessory basal nucleus |  |
| Amygdalar nuclei volume of the left anterior amygdaloid area |  |
| Amygdalar nuclei volume of the left central nucleus |  |
| Amygdalar nuclei volume of the left medial nucleus |  |
| Amygdalar nuclei volume of the left cortical nucleus |  |
| Amygdalar nuclei volume of the left cortico-amygdaloid transition area |  |
| Amygdalar nuclei volume of the left paralaminar nucleus |  |
| Amygdalar nuclei volume of the left whole amygdala |  |
| Amygdalar nuclei volume of the right lateral nucleus |  |
| Amygdalar nuclei volume of the right basal nucleus |  |
| Amygdalar nuclei volume of the right accessory basal nucleus |  |
| Amygdalar nuclei volume of the right anterior amygdaloid area |  |
| Amygdalar nuclei volume of the right central nucleus |  |
| Amygdalar nuclei volume of the right medial nucleus |  |
| Amygdalar nuclei volume of the right cortical nucleus |  |
| Amygdalar nuclei volume of the right cortico-amygdaloid transition area |  |
| Amygdalar nuclei volume of the right paralaminar nucleus |  |
| Amygdalar nuclei volume of the right whole amygdala |  |
| Thalamic nuclei volume of the left anterior ventral nucelus |  |
| Thalamic nuclei volume of the left central medial nucelus |  |
| Thalamic nuclei volume of the left central lateral nucelus |  |
| Thalamic nuclei volume of the left centromedian nucelus |  |
| Thalamic nuclei volume of the left lateral dorsal nucleus |  |
| Thalamic nuclei volume of the left lateral geniculate nucelus |  |
| Thalamic nuclei volume of the left lateral posterior nucelus |  |
| Thalamic nuclei volume of the left limitans-suprageniculate nucelus |  |
| Thalamic nuclei volume of the left mediodorsal lateral parvocellular nucelus |  |
| Thalamic nuclei volume of the left mediodorsal medial magnocellular nucelus |  |
| Thalamic nuclei volume of the left medial geniculate nucelus |  |
| Thalamic nuclei volume of the left reuniens-medial ventral nucelus |  |
| Thalamic nuclei volume of the left paracentral nucelus |  |
| Thalamic nuclei volume of the left parafascicular nucelus |  |
| Thalamic nuclei volume of the left paratenial nucelus |  |
| Thalamic nuclei volume of the left pulvinar anterior nucelus |  |
| Thalamic nuclei volume of the left pulvinar inferior nucelus |  |
| Thalamic nuclei volume of the left pulvinar lateral nucelus |  |
| Thalamic nuclei volume of the left pulvinar medial nucelus |  |
| Thalamic nuclei volume of the left ventral anterior nucelus |  |
| Thalamic nuclei volume of the left ventral anterior magnocellular nucelus |  |
| Thalamic nuclei volume of the left ventral lateral anterior nucelus |  |
| Thalamic nuclei volume of the left ventral lateral posterior nucelus |  |
| Thalamic nuclei volume of the left ventral medial nucelus |  |
| Thalamic nuclei volume of the left ventral posterior lateral nucelus |  |
| Thalamic nuclei volume of the left whole thalamus |  |
| Thalamic nuclei volume of the right anterior ventral nucelus |  |
| Thalamic nuclei volume of the right central medial nucelus |  |
| Thalamic nuclei volume of the right central lateral nucelus |  |
| Thalamic nuclei volume of the right centromedian nucelus |  |
| Thalamic nuclei volume of the right lateral dorsal nucleus |  |
| Thalamic nuclei volume of the right lateral geniculate nucelus |  |
| Thalamic nuclei volume of the right lateral posterior nucelus |  |
| Thalamic nuclei volume of the right limitans-suprageniculate nucelus |  |
| Thalamic nuclei volume of the right mediodorsal lateral parvocellular nucelus |  |
| Thalamic nuclei volume of the right mediodorsal medial magnocellular nucelus |  |
| Thalamic nuclei volume of the right medial geniculate nucelus |  |
| Thalamic nuclei volume of the right reuniens-medial ventral nucelus |  |
| Thalamic nuclei volume of the right paracentral nucelus |  |
| Thalamic nuclei volume of the right parafascicular nucelus |  |
| Thalamic nuclei volume of the right paratenial nucelus |  |
| Thalamic nuclei volume of the right pulvinar anterior nucelus |  |
| Thalamic nuclei volume of the right pulvinar inferior nucelus |  |
| Thalamic nuclei volume of the right pulvinar lateral nucelus |  |
| Thalamic nuclei volume of the right pulvinar medial nucelus |  |
| Thalamic nuclei volume of the right ventral anterior nucelus |  |
| Thalamic nuclei volume of the right ventral anterior magnocellular nucelus |  |
| Thalamic nuclei volume of the right ventral lateral anterior nucelus |  |
| Thalamic nuclei volume of the right ventral lateral posterior nucelus |  |
| Thalamic nuclei volume of the right ventral medial nucelus |  |
| Thalamic nuclei volume of the right ventral posterior lateral nucelus |  |
| Thalamic nuclei volume of the right whole thalamus |  |
| Mean cortical thickness of the left hemisphere |  |
| Mean cortical thickness of the right hemisphere |  |
| Cortical thickness of the left caudal middle frontal gyrus |  |
| Cortical thickness of the left lateral orbitofrontal gyrus |  |
| Cortical thickness of the left medial orbitofrontal gyrus |  |
| Cortical thickness of the left pars opercularis |  |
| Cortical thickness of the left pars orbitalis |  |
| Cortical thickness of the left pars triangularis |  |
| Cortical thickness of the left precentral gyrus |  |
| Cortical thickness of the left rostral middle frontal gyrus |  |
| Cortical thickness of the left superior frontal gyrus |  |
| Cortical thickness of the left frontal pole |  |
| Cortical thickness of the left bank of the superior temporal sulcus |  |
| Cortical thickness of the left entorhinal cortex |  |
| Cortical thickness of the left fusiform gyrus |  |
| Cortical thickness of the left inferior temporal gyrus |  |
| Cortical thickness of the left middle temporal gyrus |  |
| Cortical thickness of the left parahippocampal gyrus |  |
| Cortical thickness of the left superior temporal gyrus |  |
| Cortical thickness of the left temporal pole |  |
| Cortical thickness of the left transverse temporal gyrus |  |
| Cortical thickness of the left insular cortex |  |
| Cortical thickness of the left caudal anterior cingulate cortex |  |
| Cortical thickness of the left isthmus of the cingulate cortex |  |
| Cortical thickness of the left posterior cingulate cortex |  |
| Cortical thickness of the left rostral anterior cingulate cortex |  |
| Cortical thickness of the left inferior parietal gyrus |  |
| Cortical thickness of the left paracentral gyrus |  |
| Cortical thickness of the left postcentral gyrus |  |
| Cortical thickness of the left precuneus |  |
| Cortical thickness of the left superior temporal gyrus |  |
| Cortical thickness of the left supramarginal gyrus |  |
| Cortical thickness of the left cuneus |  |
| Cortical thickness of the left lateral occipital gyrus |  |
| Cortical thickness of the left lingual gyrus |  |
| Cortical thickness of the left pericalcarine gyrus |  |
| Cortical thickness of the right caudal middle frontal gyrus |  |
| Cortical thickness of the right lateral orbitofrontal gyrus |  |
| Cortical thickness of the right medial orbitofrontal gyrus |  |
| Cortical thickness of the right pars opercularis |  |
| Cortical thickness of the right pars orbitalis |  |
| Cortical thickness of the right pars triangularis |  |
| Cortical thickness of the right precentral gyrus |  |
| Cortical thickness of the right rostral middle frontal gyrus |  |
| Cortical thickness of the right superior frontal gyrus |  |
| Cortical thickness of the right frontal pole |  |
| Cortical thickness of the right bank of the superior temporal sulcus |  |
| Cortical thickness of the right entorhinal cortex |  |
| Cortical thickness of the right fusiform gyrus |  |
| Cortical thickness of the right inferior temporal gyrus |  |
| Cortical thickness of the right middle temporal gyrus |  |
| Cortical thickness of the right parahippocampal gyrus |  |
| Cortical thickness of the right superior temporal gyrus |  |
| Cortical thickness of the right temporal pole |  |
| Cortical thickness of the right transverse temporal gyrus |  |
| Cortical thickness of the right insular cortex |  |
| Cortical thickness of the right caudal anterior cingulate cortex |  |
| Cortical thickness of the right isthmus of the cingulate cortex |  |
| Cortical thickness of the right posterior cingulate cortex |  |
| Cortical thickness of the right rostral anterior cingulate cortex |  |
| Cortical thickness of the right inferior parietal gyrus |  |
| Cortical thickness of the right paracentral gyrus |  |
| Cortical thickness of the right postcentral gyrus |  |
| Cortical thickness of the right precuneus |  |
| Cortical thickness of the right superior temporal gyrus |  |
| Cortical thickness of the right supramarginal gyrus |  |
| Cortical thickness of the right cuneus |  |
| Cortical thickness of the right lateral occipital gyrus |  |
| Cortical thickness of the right lingual gyrus |  |
| Cortical thickness of the right pericalcarine gyrus |  |
